# Supplementary material for: Tissue-resident memory CD8 T cell diversity is spatiotemporally imprinted
Source: Nature. 2025 Jan 22;639(8054):483–92. doi: 10.1038/s41586-024-08466-x (PMC11903307; doi:10.1038/s41586-024-08466-x)

nature portfolio

https://doi.org/10.1038/s41586-024-08466-x

Supplementary information

Tissue-resident memory CD8 T cell diversity is spatiotemporally imprinted

In the format provided by the authors and unedited

Gating for lymphocytes

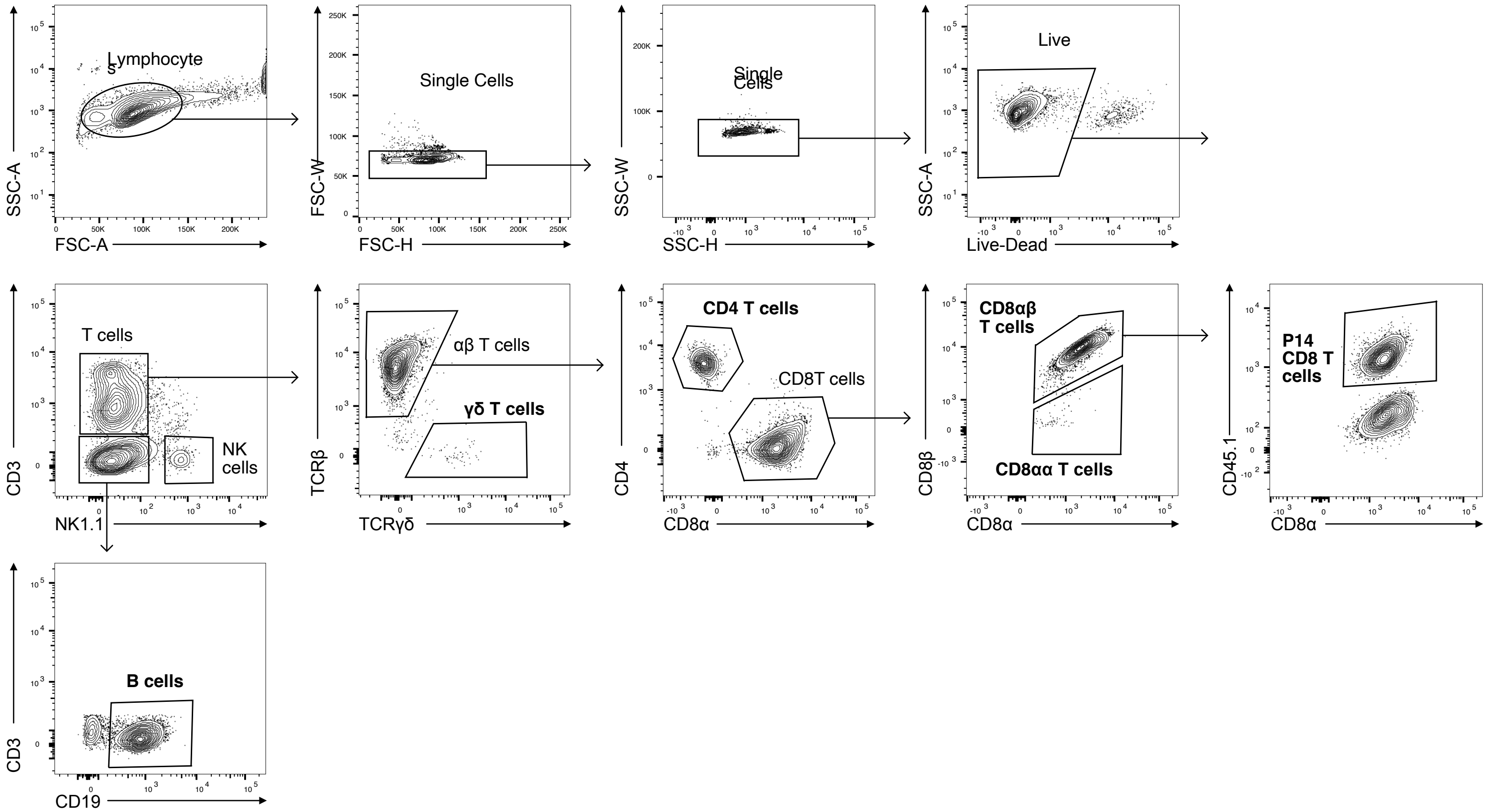

Gating for myeloid cells

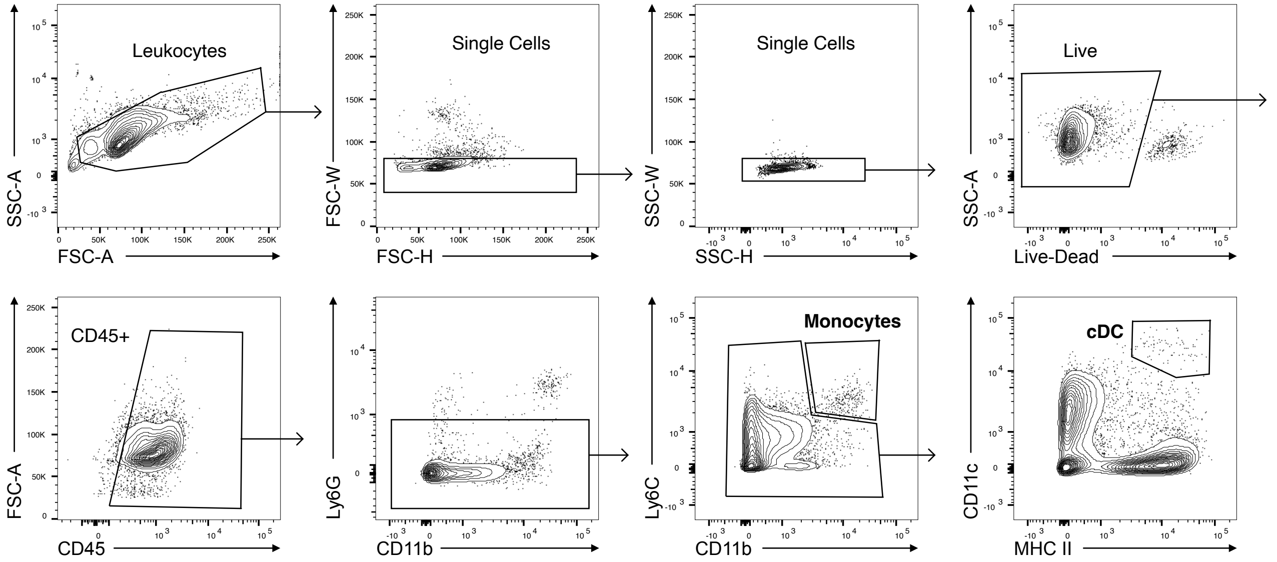

Supplement: Supplementary file 1 — The gating strategy for lymphocytes and myeloid cells in flow cytometry analysis. [file 41586_2024_8466_MOESM1_ESM.pdf]
