# Supplementary material for: Tissue-resident memory CD8 T cell diversity is spatiotemporally imprinted
Source: Nature. 2025 Jan 22;639(8054):483–92. doi: 10.1038/s41586-024-08466-x (PMC11903307; doi:10.1038/s41586-024-08466-x)
Supplement: Supplementary file 2 — Reporting Summary [file 41586_2024_8466_MOESM2_ESM.pdf]

nature portfolio

Corresponding author(s): Goldrath, Heeg

Last updated by author(s): 11/01/2024

Reporting Summary

Nature Portfolio wishes to improve the reproducibility of the work that we publish. This form provides structure for consistency and transparency in reporting. For further information on Nature Portfolio policies, see our Editorial Policies and the Editorial Policy Checklist.

Statistics

For all statistical analyses, confirm that the following items are present in the figure legend, table legend, main text, or Methods section.

n/a	Confirmed	
☐	☒	The exact sample size (*n*) for each experimental group/condition, given as a discrete number and unit of measurement
☐	☒	A statement on whether measurements were taken from distinct samples or whether the same sample was measured repeatedly
☐	☒	The statistical test(s) used AND whether they are one- or two-sided<br>*Only common tests should be described solely by name; describe more complex techniques in the Methods section.*
☐	☒	A description of all covariates tested
☐	☒	A description of any assumptions or corrections, such as tests of normality and adjustment for multiple comparisons
☐	☒	A full description of the statistical parameters including central tendency (e.g. means) or other basic estimates (e.g. regression coefficient) AND variation (e.g. standard deviation) or associated estimates of uncertainty (e.g. confidence intervals)
☐	☒	For null hypothesis testing, the test statistic (e.g. *F*, *t*, *r*) with confidence intervals, effect sizes, degrees of freedom and *P* value noted<br>*Give P values as exact values whenever suitable.*
☒	☐	For Bayesian analysis, information on the choice of priors and Markov chain Monte Carlo settings
☒	☐	For hierarchical and complex designs, identification of the appropriate level for tests and full reporting of outcomes
☐	☒	Estimates of effect sizes (e.g. Cohen's *d*, Pearson's *r*), indicating how they were calculated

Our web collection on statistics for biologists contains articles on many of the points above.

Software and code

Policy information about availability of computer code

Data collection

Histology and Immunofluorescence images were acquired on an Olympus VS200 Slide Scanner (UCSD Microscopy CORE) or on a ZEISS LSM700 confocal microscope.
Spatial transcriptomics data was acquired on the 10x Xenium and Vizgen MERSCOPE.

Data analysis

Data processing pipelines and the code to reproduce all figures for this project will be made publicly available on Github (https://github.com/Goldrathlab/Spatial-TRM-paper) after publication.

The following packages have been used:

Cellpose 2.2.3
Baysor 0.6.2
MERLIN 232.230125.316b
scipy 1.10.1
torch 2.1.0
labelme 5.5.0
tensorflow 2.15.0

[dependencies]
scanpy = "==1.9.5"
scikit-learn = "==1.1.3"
scvi-tools = "==1.1.1"

```
tqdm = ">=4.66.5,<5"
r = ">=4.3,<4.4"
r-tidyverse = ">=2.0.0,<3"
jupyterlab = ">=4.2.5,<5"
r-irkernel = ">=1.3.2,<2"
bioconductor-zellkonverter = ">=1.12.1,<2"
radian = ">=0.6.13,<0.7"
bioconductor-genomeinfodbdata = ">=1.2.11,<2"
r-biocmanager = ">=1.30.25,<2"
bioconductor-scater = ">=1.30.1,<2"
bioconductor-scran = ">=1.30.0,<2"
bioconductor-scuttle = ">=1.12.0,<2"
bioconductor-singlecellexperiment = ">=1.24.0,<2"
imageio = ">=2.35.1,<3"
alphashape = ">=1.3.1,<2"
opencv = ">=4.10.0,<5"
tifffile = ">=2024.8.28,<2025"
ipywidgets = ">=8.1.5,<9"
pre_commit = ">=3.8.0,<4"
openpyxl = ">=3.1.5,<4"
scvelo = "==0.3.1"
squidpy = ">=1.2.3,<2"
optax = "0.2.0.*"
libegl = ">=1.7.0,<2"
libopengl = ">=1.7.0,<2"
libgl = ">=1.7.0,<2"
python = ">=3.10.14,<3.11"
r-remotes = ">=2.5.0,<3"
bioconductor-complexheatmap = ">=2.18.0,<3"
r-lme4 = ">=1.1_35.5,<2"
r-future = ">=1.34.0,<2"
r-patchwork = ">=1.2.0,<2"
r-svglite = ">=2.1.3,<3"
r-rstatix = ">=0.7.2,<0.8"
r-shiny = ">=1.9.1,<2"
r-pbkrtest = ">=0.5.3,<0.6"
r-plotly = ">=4.10.4,<5"
r-ggsci = ">=3.2.0,<4"
r-ggpubr = ">=0.6.0,<0.7"
r-pbapply = ">=1.7_2,<2"
r-nmf = ">=0.21.0,<0.22"
r-ggalluvial = ">=0.12.5,<0.13"
r-network = ">=1.18.2,<2"
r-ggnetwork = ">=0.5.13,<0.6"
r-rspectra = ">=0.16_2,<0.17"
r-presto = ">=1.0.0,<2"

[pypi-dependencies]
ucell = { git = "https://github.com/maximilian-heeg/UCell.git", rev = "3d29122" }
```

For manuscripts utilizing custom algorithms or software that are central to the research but not yet described in published literature, software must be made available to editors and reviewers. We strongly encourage code deposition in a community repository (e.g. GitHub). See the Nature Portfolio guidelines for submitting code & software for further information.

Data

Policy information about availability of data

All manuscripts must include a data availability statement. This statement should provide the following information, where applicable:

- Accession codes, unique identifiers, or web links for publicly available datasets
- A description of any restrictions on data availability
- For clinical datasets or third party data, please ensure that the statement adheres to our policy

The code to reproduce the analysis presented in this manuscript is available on GitHub (https://github.com/Goldrathlab/Spatial-TRM-paper). Sequencing data and spatial transciptomics data are deposited in GEO: GSE279254 (VisiumHD), GSE279255 (snRNA sequencing) and GSE280895 (Xenium & Merscope).

Research involving human participants, their data, or biological material

Policy information about studies with human participants or human data. See also policy information about sex, gender (identity/presentation), and sexual orientation and race, ethnicity and racism.

Reporting on sex and gender: 2/2 healthy donors were female.

Reporting on race, ethnicity, or other socially relevant groupings	No information about ethnicity or race available.
Population characteristics	Both healthy donors were adults, no further age characteristics are available.
Recruitment	Deidentified human FFPE samples from healthy subjects were acquired from the San Diego Digestive Diseases Research Center (SDDRC).
Ethics oversight	The Human Research Protection Programs at the University of California, San Diego reviewed and approved the protocol, including a waiver of consent.

Note that full information on the approval of the study protocol must also be provided in the manuscript.

Field-specific reporting

Please select the one below that is the best fit for your research. If you are not sure, read the appropriate sections before making your selection.

☒ Life sciences ☐ Behavioural & social sciences ☐ Ecological, evolutionary & environmental sciences

For a reference copy of the document with all sections, see nature.com/documents/nr-reporting-summary-flat.pdf

Life sciences study design

All studies must disclose on these points even when the disclosure is negative.

Sample size	No statistical methods were used to pre-determine sample sizes, but our sample sizes are like those reported in previous publications (e.g. Milner et al, Nature 2017) from our laboratory and others.
Data exclusions	No data were excluded
Replication	All mouse experiments were successfully repeated ≥2 times and where possible quantification and statistics were run on combined replicate experiments, when possible.
Randomization	All mice were between 1.5 and 6 months old at the time of infection and randomly assigned to experimental groups. Human samples were not randomized.
Blinding	No blinding was performed during mouse experiments. The experimental observations presented would be consistent irrespective of blinding and therefore blinding was not relevant in this study.

Reporting for specific materials, systems and methods

We require information from authors about some types of materials, experimental systems and methods used in many studies. Here, indicate whether each material, system or method listed is relevant to your study. If you are not sure if a list item applies to your research, read the appropriate section before selecting a response.

Materials & experimental systems

n/a	Involved in the study	
☐	☒	Antibodies
☒	☐	Eukaryotic cell lines
☒	☐	Palaeontology and archaeology
☐	☒	Animals and other organisms
☒	☐	Clinical data
☒	☐	Dual use research of concern
☒	☐	Plants

Methods

n/a	Involved in the study	
☒	☐	ChIP-seq
☐	☒	Flow cytometry
☒	☐	MRI-based neuroimaging

Antibodies

Antibodies used

Marker Color Clone # Vendor Catalog # Dilution Validation
CD3 PE 145-2C11 eBioscience 12-0031-83 200 flow cytometric analysis of mouse thymocytes and splenocytes
TCR ab APC H57-597 eBioscience 17-5961-83 200 flow cytometric analysis of mouse thymocytes and splenocytes
NK1.1 FITC PK136 eBioscience 11-5941-81 400 flow cytometric analysis of C57Bl/6 mouse splenocytes
CD19 PerCP-Cy5.5 eBio1D3 eBioscience 45-0193-82 200 flow cytometric analysis of mouse splenocytes
CD8b BV421 H35-17.2 eBioscience 48-0083-82 400 flow cytometric analysis of mouse splenocytes
CD45.1 BV510 A20 Biolegend 110741 200 Verified Reactivity: Mouse

TCR gd BV711 GL3 Biolegend 118149 200 Verified Reactivity: Mouse
CD4 BV786 GK1.5 Biolegend 100453 400 Verified Reactivity: Mouse
CD8a PE-Cy7 53-6.7 eBioscience 25-0081-82 400 flow cytometric analysis of mouse thymocytes and splenocytes
CD11b PE M1/70 eBioscience 12-0112-82 200 flow cytometric analysis of mouse splenocytes or bone marrow cells
CD11c APC N418 Biolegend 117310 200 Verified Reactivity: Mouse
Ly6C FITC AL-21 BD 553104 200 "Reactivity: Mouse (QC Testing)
Application:Flow cytometry (Routinely Tested)"
Ly6G PerCP-Cy5.5 1A8 Biolegend 127615 200 Verified Reactivity: Mouse
Xcr1 BV421 ZET Biolegend 148216 200 Verified Reactivity: Mouse, Rat
CD45 BV510 30-F11 BD 561487 200 "Reactivity: Mouse (QC Testing)
Application:Flow cytometry (Routinely Tested)"
F4/80 BV711 BM8 Biolegend 123147 200 Verified Reactivity: Mouse
MHC II BV786 M5/114.15.2 Biolegend 107645 200 Verified Reactivity: Mouse
B220 PE-Cy7 RA3-6 B2 Biolegend 103222 200 "Verified Reactivity: Mouse, Human
Reported Reactivity: Cat"
Fixable Viability Dye APC-Cy7 eBioscience 65-0865-14 1000 flow cytometric analysis of mouse thymocytes
anti-hamster IgG n/a polyclonal Thermo Fisher Scientific PI31115 50
CD3e n/a 145-2C11 Fisher Scientific 50-112-9591 1000 flow cytometric analysis of mouse splenocytes
CD28 n/a 37.51 Fisher Scientific 50-112-9711 1000 flow cytometric analysis of mouse splenocytes
Thy1.2 BV510 30-H12 BioLegend 105335 200 flow cytometric analysis of mouse splenocytes
Cxcr3 APC Cxcr3-173 eBiosciences 17-1831-82 200 flow cytometric analysis of mouse splenocytes
CD45.1 AF594 A20 BioLegend 110756 50 IHC-F - Quality tested
E-cadherin APC DECMA-1 BioLegend 147312 200 FC - Quality tested
CD8a FITC 53-6.7 cytekbio 35-0081-U500 50 Flow cytometry
CD8a n/a/ EPR21769 abcam ab217344 50 Suitable for IP, Flow Cyt, WB, IHC-Fr, IHC-P and reacts with Mouse samples
Anti-rabbit AF594 Polyclonal Invitrogen A-11012 200

Validation

All antibodies were obtained from commercial vendors.
Marker Color Clone # Vendor Catalog # Dilution Validation
CD3 PE 145-2C11 eBioscience 12-0031-83 200 flow cytometric analysis of mouse thymocytes and splenocytes
TCR ab APC H57-597 eBioscience 17-5961-83 200 flow cytometric analysis of mouse thymocytes and splenocytes
NK1.1 FITC PK136 eBioscience 11-5941-81 400 flow cytometric analysis of C57Bl/6 mouse splenocytes
CD19 PerCP-Cy5.5 eBio1D3 eBioscience 45-0193-82 200 flow cytometric analysis of mouse splenocytes
CD8b BV421 H35-17.2 eBioscience 48-0083-82 400 flow cytometric analysis of mouse splenocytes
CD45.1 BV510 A20 Biolegend 110741 200 Verified Reactivity: Mouse
TCR gd BV711 GL3 Biolegend 118149 200 Verified Reactivity: Mouse
CD4 BV786 GK1.5 Biolegend 100453 400 Verified Reactivity: Mouse
CD8a PE-Cy7 53-6.7 eBioscience 25-0081-82 400 flow cytometric analysis of mouse thymocytes and splenocytes
CD11b PE M1/70 eBioscience 12-0112-82 200 flow cytometric analysis of mouse splenocytes or bone marrow cells
CD11c APC N418 Biolegend 117310 200 Verified Reactivity: Mouse
Ly6C FITC AL-21 BD 553104 200 "Reactivity: Mouse (QC Testing)
Application:Flow cytometry (Routinely Tested)"
Ly6G PerCP-Cy5.5 1A8 Biolegend 127615 200 Verified Reactivity: Mouse
Xcr1 BV421 ZET Biolegend 148216 200 Verified Reactivity: Mouse, Rat
CD45 BV510 30-F11 BD 561487 200 "Reactivity: Mouse (QC Testing)
Application:Flow cytometry (Routinely Tested)"
F4/80 BV711 BM8 Biolegend 123147 200 Verified Reactivity: Mouse
MHC II BV786 M5/114.15.2 Biolegend 107645 200 Verified Reactivity: Mouse
B220 PE-Cy7 RA3-6 B2 Biolegend 103222 200 "Verified Reactivity: Mouse, Human
Reported Reactivity: Cat"
Fixable Viability Dye APC-Cy7 eBioscience 65-0865-14 1000 flow cytometric analysis of mouse thymocytes
anti-hamster IgG n/a polyclonal Thermo Fisher Scientific PI31115 50
CD3e n/a 145-2C11 Fisher Scientific 50-112-9591 1000 flow cytometric analysis of mouse splenocytes
CD28 n/a 37.51 Fisher Scientific 50-112-9711 1000 flow cytometric analysis of mouse splenocytes
Thy1.2 BV510 30-H12 BioLegend 105335 200 flow cytometric analysis of mouse splenocytes
Cxcr3 APC Cxcr3-173 eBiosciences 17-1831-82 200 flow cytometric analysis of mouse splenocytes
CD45.1 AF594 A20 BioLegend 110756 50 IHC-F - Quality tested
E-cadherin APC DECMA-1 BioLegend 147312 200 FC - Quality tested
CD8a FITC 53-6.7 cytekbio 35-0081-U500 50 Flow cytometry
CD8a n/a/ EPR21769 abcam ab217344 50 Suitable for IP, Flow Cyt, WB, IHC-Fr, IHC-P and reacts with Mouse samples
Anti-rabbit AF594 Polyclonal Invitrogen A-11012 200

Animals and other research organisms

Policy information about studies involving animals; ARRIVE guidelines recommended for reporting animal research, and Sex and Gender in Research

Laboratory animals

Mice were maintained in specific-pathogen-free conditions at a temperature between 18°C and 23°C with 40–60% humidity and a 12h-light and 12h-dark light cycle in accordance with the Institutional Animal Care and Use Committees (IACUC) of the University of California San Diego (UCSD). All mice were of C57BL/6J background and bred at the University of California San Diego (UCSD) or purchased from the Jackson Laboratory. R26Cre-ERT2 (stock no. 008463, Jackson Laboratory), Tgfbr2fl/fl (stock no. 012603, Jackson Laboratory), P14, and CD45.1 congenic mice were bred in-house. All mice used were between 1.5 and 6 months old.

Wild animals	No wild animals were used.
Reporting on sex	For immunofluorescence male and female mice were used for infection experiments and adoptively transferred P14 cells were either sex matched or male cells were transferred into female recipients.<br>For spatial transcriptomic experiments male TCR-transgenic T cells were adoptively transferred into female C57BL/6 recipient mice.
Field-collected samples	The study did not involve samples collected from the field.
Ethics oversight	Institutional Animal Care and Use Committee of the University of California San Diego

Note that full information on the approval of the study protocol must also be provided in the manuscript.

Plants

Seed stocks	*Report on the source of all seed stocks or other plant material used. If applicable, state the seed stock centre and catalogue number. If plant specimens were collected from the field, describe the collection location, date and sampling procedures.*
Novel plant genotypes	*Describe the methods by which all novel plant genotypes were produced. This includes those generated by transgenic approaches, gene editing, chemical/radiation-based mutagenesis and hybridization. For transgenic lines, describe the transformation method, the number of independent lines analyzed and the generation upon which experiments were performed. For gene-edited lines, describe the editor used, the endogenous sequence targeted for editing, the targeting guide RNA sequence (if applicable) and how the editor was applied.*
Authentication	*Describe any authentication procedures for each seed stock used or novel genotype generated. Describe any experiments used to assess the effect of a mutation and, where applicable, how potential secondary effects (e.g. second site T-DNA insertions, mosiacism, off-target gene editing) were examined.*

Flow Cytometry

Plots

Confirm that:

☒ The axis labels state the marker and fluorochrome used (e.g. CD4-FITC).

☒ The axis scales are clearly visible. Include numbers along axes only for bottom left plot of group (a 'group' is an analysis of identical markers).

☒ All plots are contour plots with outliers or pseudocolor plots.

☒ A numerical value for number of cells or percentage (with statistics) is provided.

Methodology

Sample preparation	Isolation of CD8 T cells was performed similarly as described (Steinert at al, 2015). Small intestine (SI) intra-epithelial lymphocytes (IEL) and lamina propria lymphocytes (LPL) were prepared by removing Peyer's patches and the luminal contents from the entire SI. The SI was then cut longitudinally and into 1 cm pieces, then incubated at 37°C for 30 minutes in HBSS with 2.1 mg/mL sodium bicarbonate, 2.4 mg/mL HEPES, 8% bovine growth serum, and 0.154 mg/mL of dithioerythritol (EMD Millipore). Collection of the supernatant through a 70 µM constituted the IEL compartment of the SI. The remaining tissue fragments of the SI were further incubated in RPMI with 1.2 mg/mL HEPES, 292 µ/mL L-glutamine, 1 mM MgCl2, 1 mM CaCl2, 5% fetal bovine serum, and 100 U/mL collagenase (Worthington) at 37°C for 30 min. After enzymatic incubation, tissues were filtered through a 70-µm nylon cell strainer (Falcon). Tissue preparations were separated on a 44%/67% Percoll density gradient.
Instrument	For flow cytometry, all events were acquired on a BD LSRFortessa X-20 or a BD LSRFortessa.
Software	FlowJo v10.10.0
Cell population abundance	Sorted transduced samples had a purity of > 95%.
Gating strategy	The gating strategy is shown in supplemental figure 1.

☒ Tick this box to confirm that a figure exemplifying the gating strategy is provided in the Supplementary Information.
