# Supplementary material for: Tissue-resident memory CD8 T cell diversity is spatiotemporally imprinted
Source: Nature. 2025 Jan 22;639(8054):483–92. doi: 10.1038/s41586-024-08466-x (PMC11903307; doi:10.1038/s41586-024-08466-x)
Supplement: Supplementary file 4 — Supplementary Tables 1–12 [file 41586_2024_8466_MOESM4_ESM.zip › New folder/2024-02-04343D-SI_Guide.docx]

**Manuscript 2024-02-04343D**

**Supplemental Information Guide**

Supplemental Table 1. Xenium custom mouse 350 gene panel

Supplemental Table 2. QC timecourse biological duplicate statistics

Supplemental Table 3. Gene expression correlation by subtype and spatial axes

Supplemental Table 4. L-R pathway contributions for the LCMV time course

Supplemental Table 5. L-R pathway contributions for the VisiumHD dataset

Supplemental Table 6. Xenium custom mouse 480 gene panel

Supplemental Table 7. Merscope 494 custom mouse gene panel

Supplemental Table 8. KS statistics of cell distance analysis

Supplemental Table 9. sgRNA sequences

Supplemental Table 10. Xenium custom human gene panel

Supplemental Table 11. Number of perturbed cells across spatial gates

Supplemental Table 12. L-R pathway contributions for the human ileum dataset
